# *EFG1* Mutations, Phenotypic Switching, and Colonization by Clinical a/α Strains of Candida albicans

**DOI:** 10.1128/mSphere.00795-19

**Published:** 2020-02-05

**Authors:** Yang-Nim Park, Kayla Conway, Claude Pujol, Karla J. Daniels, David R. Soll

**Affiliations:** a Developmental Studies Hybridoma Bank, Department of Biology, The University of Iowa, Iowa City, Iowa, USA; University of Georgia

**Keywords:** *Candida albicans*, *EFG1* mutation, clinical isolates, switching to opaque

## Abstract

The transcription factor *EFG1* functions as a suppressor of white-to-opaque and white-to-gray switching in **a**/α strains of Candida albicans. In a collection of 27 clinical isolates, 4 of the 17 *EFG1/EFG1* strains, 1 of the 2 *EFG1/efg1* strains, and all 8 of the *efg1*/*efg1* strains underwent white-to-opaque switching. The four *EFG1/EFG1* strains, the one *EFG1/efg1* strain, and one of the eight *efg1*/*efg1* strains that underwent switching to opaque did not switch to gray and could not be complemented with a copy of *EFG1*. Competition experiments in a mouse model for gastrointestinal (GI) colonization confirmed that *efg1*/*efg1* cells rapidly outcompete *EFG1*/*EFG1* cells, and in plating experiments, formed colonies containing both gray and opaque cells. Direct microscopic analysis of live cells in the feces, however, revealed that the great majority of cells were opaque, suggesting opaque, not gray, may be the dominant phenotype at the site of colonization.

**IMPORTANCE** Close to half of a collection of 27 clinical **a**/α isolates of Candida albicans underwent white-to-opaque switching. Complementation experiments revealed that while approximately half of the **a**/α switchers were due to *EFG1* mutations, the remaining half were due to mutations in other genes. In addition, the results of competition experiments in a mouse GI tract colonization model support previous observations that *efg1*/*efg1* cells rapidly outcompete *EFG1/EFG1* strains, but direct microscopic analysis reveals that the major colonizing cells were opaque, not gray.

## Abstract (precis)

Close to half of a collection of 27 clinical **a**/α isolates of Candida albicans underwent white-to-opaque switching. Complementation experiments revealed that while approximately half of the **a**/α switchers were due to *EFG1* mutations, the remaining half were due to mutations in other genes. In addition, the results of competition experiments in a mouse GI tract colonization model support previous observations that *efg1*/*efg1* cells rapidly outcompete *EFG1/EFG1* strains, but direct microscopic analysis reveals that the major colonizing cells were opaque, not gray.

## INTRODUCTION

Candida albicans remains a pervasive opportunistic fungal pathogen, colonizing a majority of humans as a commensal (1). It was initially believed that its success as an opportunistic pathogen was due in part to its capacity to invade tissue through a single developmental program, the transition from a budding yeast to a filamentous hypha (1, 2). However, in 1987, a second reversible phenotypic transition, the white-opaque switching system, was identified in a strain of C. albicans, WO-1, isolated from a bone marrow transplant patient (3, 4). Soon after, it was reported that only select strains of C. albicans underwent the white-opaque transition (5). Switching affected not only colony morphology but also impacted most aspects of cell morphology and cell wall architecture (3, 6, 7). In the 15 years after the discovery of white-opaque switching, unique patterns of differential gene expression were described (8–14), and differences in virulence were demonstrated in mouse models of skin colonization (15) and systemic colonization (16). However, the role of switching remained elusive. That appeared to end in 2002, when Miller and Johnson (17) discovered that the configuration of the mating type locus (*MTL*) regulated switching and switching in turn regulated mating. They discovered that hemizygous *MTL* derivatives of a heterozygous *MTL* (**a**/α) strain, the latter representing the predominant *MTL* genotype among clinical isolates (18, 19), could switch. This led to the conclusion that minority **a/a** and α/α strains could switch, but majority **a**/α strains could not (19). This perception, however, ended when Xie et al. (20) reported that approximately one third of a collection of clinical **a**/α isolates could be induced to switch on agar medium containing *N*-acetylglucosamine (GlcNAc) at 25°C in 5% CO_2_. Furthermore, Xie et al. (20) demonstrated that individually deleting any one of the genes for the transcription factors (TFs) Efg1, Rfg1, or Brg1, derepressed switching to opaque in **a**/α cells, and Park et al. (21) subsequently demonstrated that deletion of the TF gene *SFL2* also derepressed switching in **a**/α strains. Tao et al. (22) subsequently demonstrated that select **a**/α clinical isolates that underwent white-opaque switching could also switch to a “gray” phenotype, and this was confirmed by Park et al. (21), suggesting the existence of a triphasic switching system. Liang et al. (23) further presented evidence that clinical **a**/α strains that underwent switching to gray harbored a mutation in *EFG1*, and that, as previously demonstrated by Pierre and Kumamoto (24), **a**/α *EFG1* mutants outcompeted wild-type cells in a mouse gastrointestinal (GI) colonization model. Using a CHROMagar plating assay, Liang et al. (23) further presented evidence indicating that the colonizing *EFG1* mutant cells expressed the gray phenotype, although Park et al. (21) presented evidence that the gray phenotypes did not appear to be expressed by **a**/α *EFG1* mutant cells at physiological temperature (37°C).

Here, we have explored the role of *EFG1* in white-to-opaque or white-to-gray switching, and GI colonization, in a collection of 27 clinical **a**/α isolates. We found that 13 of the 27 isolates could be induced to switch to opaque. Four of the 17 *EFG1/EFG1* strains, 1 of the 2 *EFG1*/*efg1* strains, and all 8 of the *efg1*/*efg1* strains underwent switching from white to opaque. However, only 7 of the 13 **a**/α isolates that switched were complemented by site-specific integration of a copy of *EFG1*, and all 7 were *efg1*/*efg1* mutants. In all cases in which complementation with *EFG1* reestablished repression of white-opaque switching, it also reestablished repression of white-gray switching. These results demonstrate that mutants of genes that repress white-to-opaque switching other than *EFG1* may be just as prevalent as *EFG1* mutants in clinical **a**/α strains that switch. Finally, results from competition experiments in a mouse gastrointestinal colonization model confirmed previous results (23, 24) that *efg1Δ*/*efg1Δ* cells or *efg1*/*efg1* cells outcompete *EFG1/EFG1* cells. When we plated the cells that colonized the GI tract, the results suggested that they formed gray and opaque cells. However, direct microscopic observations of live colonizing cells in the feces revealed that **a**/α strains with *EFG1* null mutations expressed almost exclusively the opaque, not gray, phenotype, suggesting plating experiments may not accurately assess the cell phenotypes expressed at the site of GI colonization. Therefore, the latter results suggest that **a**/α *efg1*/*efg1* strains colonizing the GI tract may express the opaque, not gray, phenotype.

## RESULTS

### *EFG1* deletion mutants of strains SC5314 and P37039.

**FIG 1:**
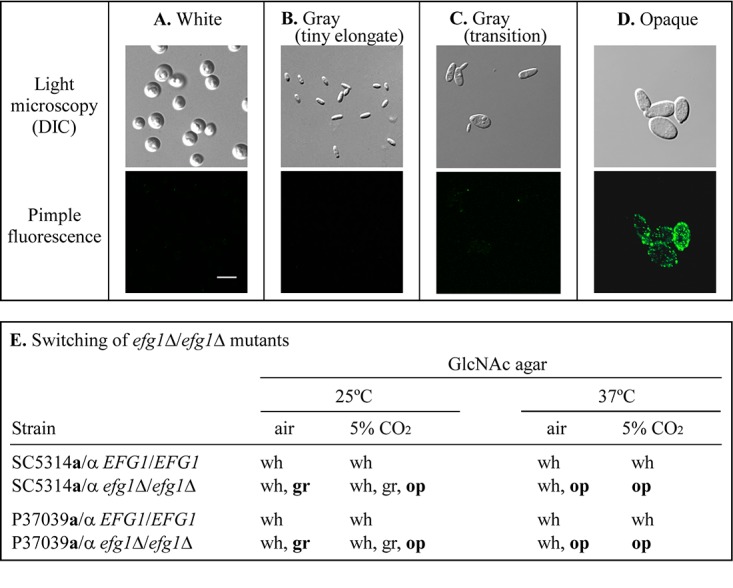
Examples of **a**/α cellular phenotype assessed in the switching studies and a summary of switching by the two strains SC5314 and P37039 and their deletion derivatives, previously analyzed in detail (21). Note that “tiny elongate” and “transition” represent two subgroups of the gray phenotype. (A) White phenotype; (B) gray tiny elongate phenotype; (C) gray transition phenotype; (D) opaque phenotype. In panels A to D, a differential interference contrast (DIC) image is presented in the top panel, and the corresponding immunostained image, with anti-opaque-specific pimple antibody, is shown in the bottom panel. (E) The phenotypes of the two parental **a**/α strains and deletion derivatives are presented under the four tested sets of environmental conditions on GlcNAc-agar. Cell phenotypes in boldface type in panel E are the dominant phenotypes formed in mixtures. wh, white; gr, gray; op, opaque. Bar in panel A, 5 μm.

**FIG 2:**
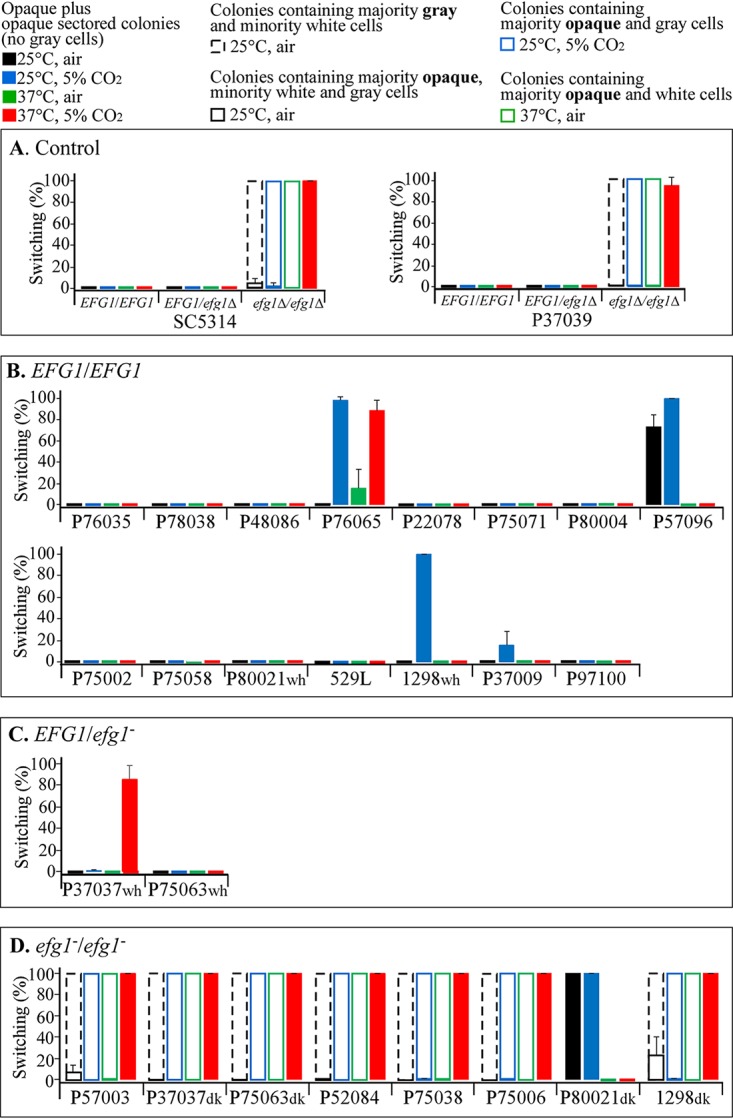
Switching to opaque and gray by the 27 clinical isolates and the generated deletion derivatives of C. albicans strains SC5314 and P37039 on GlcNAc-agar under the four sets of environmental conditions (25°C and air, 25°C and 5% CO_2_, 37°C and air, and 37°C and 5% CO_2_). The bars are explained in the key at the top of the figure. The majority cell phenotypes in the mixtures in the key are shown in boldface type. (A) Parent **a**/α SC5314 and P37039 *EFG1/EFG1* strains and their heterozygous *EFG1*/*efg1*Δ and homozygous *efg1*Δ/*efg1*Δ mutant derivatives. (B) Fifteen additional clinical *EFG1/EFG1* strains. (C) Two clinical *EFG1*/*efg1* strains. (D) Eight clinical *efg1*/*efg1* strains.

To assess the role of *EFG1* in switching in **a**/α strains, we previously generated homozygous *EFG1* deletion mutants of two **a**/α *EFG1/EFG1* strains that did not switch, SC5314 and P37009 (see Table S1 in the supplemental material), and analyzed them for switching from white to opaque and white to gray on agar media under eight sets of environmental conditions (21). The conditions included all combinatorial permutations of the sugar source (agar with 1.25% glucose [Gluc-agar] versus 2.0% *N*-acetylglucosamine [GlcNAc-agar]), temperature (25°C versus 37°C) and atmosphere (air [0.04% CO_2_] versus 5% CO_2_) (21) in supplemented Lee’s medium (25). In our previous study (21), the four cellular phenotypes that were distinguished included the original white cell phenotype (Fig. 1A), the gray phenotype, which included the tiny elongate phenotype (Fig. 1B) and the transition phenotype (Fig. 1C), and the elongate, pimpled opaque cell phenotype (Fig. 1D). On both Gluc-agar and GlcNAc-agar at 25°C or 37°C, in air or 5% CO_2_, white cells of the SC3514 and P37039 **a**/α parent strains, which were *EFG1/EFG1*, did not switch. On Gluc-agar at 25°C or 37°C, in air or 5% CO_2_, white cells of both *efg1Δ/efg1Δ* derivatives of strains SC5314 and P37039 also did not switch to the gray or opaque phenotype (21). However, on GlcNAc-agar under all four sets of environmental conditions, the two *efg1Δ/efg1Δ* strains switched to either gray, opaque and gray, or opaque. On GlcNAc agar at 25°C in air, both *efg1Δ/efg1Δ* strains formed colonies containing a majority of gray cells and a minority of white cells, but no opaque cells (Fig. 1E and 2A). On GlcNAc-agar at 25°C in 5% CO_2_, both *efg1Δ/efg1Δ* strains formed colonies containing a majority of opaque cells and a minority of white and gray cells (Fig. 1E and 2A). On GlcNAc-agar at 37°C in air, *efg1Δ/efg1Δ* strains formed colonies containing a majority of opaque cells and a minority of white cells but no gray cells (Fig. 1E and 2A). On GlcNAc-agar at 37°C in 5% CO_2_, *efg1Δ/efg1Δ* strains formed opaque colonies containing exclusively opaque cells (Fig. 1E and 2A).

10.1128/mSphere.00795-19.2 TABLE S1 Strains used in this study. Download Table S1, DOCX file, 0.02 MB. Copyright © 2020 Park et al. 2020 Park et al. This content is distributed under the terms of the Creative Commons Attribution 4.0 International license.

### Collection of clinical isolates.

The 27 clinical **a**/α isolates in the collection (Table S1) have been analyzed by restriction fragment length polymorphisms (RFLPs) probed with the mid-repeat sequence (Ca3 and by multilocus sequence typing [MLST]) as explained in the legend for Table S2. Clades I, SA, E, and III, determined by Ca3 fingerprinting, correlate with clades 1, 4, 3, and 11, determined by MLST (Table S2). The collection, based on the consensus of the clade analyses, grouped 8 in clade SA (MLST 4), 1 in clade III (MLST 3), 5 in clade E (MLST 11), and 10 in clade I (MLST 1) (Table S2). Four isolates were not tested by probed RFLPs (Table S2). One proved to be in MLST clade 3, two were in MLST 18, and one remained untested. Eight of the 27 isolates represented four pairs, each pair isolated from one of four individuals (P37037wh and P37037dk, P75063wh and P75063dk, P80021wh and P80021dk, and 1298wh and 1298dk). Using multilocus sequence typing (10, 11), we found that two strains in each of three pairs (P37037wh and P37037dk, P75063wh and P75063dk, and 1298wh and 1298dk) were highly related. The coisolates in the fourth pair (P80021wh and P80021dk) were not related (Table S2). The coisolates in each of the four pairs were treated individually in this study.

10.1128/mSphere.00795-19.3 TABLE S2 Ca3/MLST clade assignments for the 27 isolates used in this study. Ca3 fingerprinting was performed in the Soll lab. The MLST analysis and clade assignment of 10 strains were from collaboration (M.-E. Bougnoux, C. Pujol, D. Diogo, C. Bouchier, et al., Fungal Genet Biol 45:221–231, 2008, https://doi.org/10.1016/j.fgb.2007.10.008) or from different groups (F. C. Odds, M.-E. Bougnoux, D. J. Shaw, J. M. Bain, et al., Eukaryot Cell 6:1041–1052, 2007, https://doi.org/10.1128/EC.00041-07; M. P. Hirakawa, D. A. Martinez, S. Sakthikumar, M. Z. Anderson, et al., Genome Res 5:413–425, 2015, https://doi.org/10.1101/gr.174623.114). MLST typing of isolates P75063wh, P75063dk, P80021wh, P80021dk, 1298wh, 1298dk, P37037wh, and P37037dk was performed in our lab according to the consensus MLST scheme described by Bougnoux et al. (M.-E. Bougnoux, A. Tavanti, C. Bouchier, N. A. R. Gow, et al., J Clin Microbiol 41:5265–5266, 2003, https://doi.org/10.1128/JCM.41.11.5265-5266.2003) which is commonly used worldwide. The MLST clade assignment for these isolates was performed by generating a dendrogram, including MLST data retrieved from the Candida albicans MLST website (https://pubmlst.org/calbicans/) for 24 control isolates and strain 529L, which does not appear to have been previously assigned to a clade. The control isolates were selected, three per clade, from the seven most frequent clades (MLST clades 1, 2, 3, 4, 8, 9, and 11) found among a collection of 1,391 isolates (F. C. Odds, M.-E. Bougnoux, D. J. Shaw, J. M. Bain, et al., Eukaryot Cell 6:1041–1052, 2007, https://doi.org/10.1128/EC.00041-07). Three isolates from clade 18, a major clade in Asia (J. E. Shin, M.-E. Bougnoux, C. d’Enfert, S. H. Kim, et al., J Clin Microbiol 49:2572–2577, 2011, https://doi.org/10.1128/JCM.02153-10), were also used as controls. 529L did not cluster with any other strain. The Ca3 fingerprinting clades I, II, III, SA, and E have been repeatedly shown to correspond nearly perfectly to MLST clades 1, 2, 3, 4, and 11, respectively (A. Tavanti, A. D. Davidson, M. J. Fordyce, N. A. R. Gow, et al., J Clin Microbiol 43:5601–5613, 2005, https://doi.org/10.1128/JCM.43.11.5601-5613.2005; M.-E. Bougnoux, C. Pujol, D. Diogo, C. Bouchier, et al., Fungal Genet Biol 45:221–231, 2008, https://doi.org/10.1016/j.fgb.2007.10.008; F. C. Odds, M.-E. Bougnoux, D. J. Shaw, J. M. Bain, et al., Eukaryot Cell 6:1041–1052, 2007, https://doi.org/10.1128/EC.00041-07; B. A. McManus and D. C. Coleman, Infect Genet Evol 21:166–178, 2014, https://doi.org/10.1016/j.meegid.2013.11.008). It should be noted that clade assignments are no longer displayed on the C. albicans MLST website. Download Table S2, DOCX file, 0.01 MB. Copyright © 2020 Park et al. 2020 Park et al. This content is distributed under the terms of the Creative Commons Attribution 4.0 International license.

### *EFG1* mutations among the collection.

**FIG 3:**
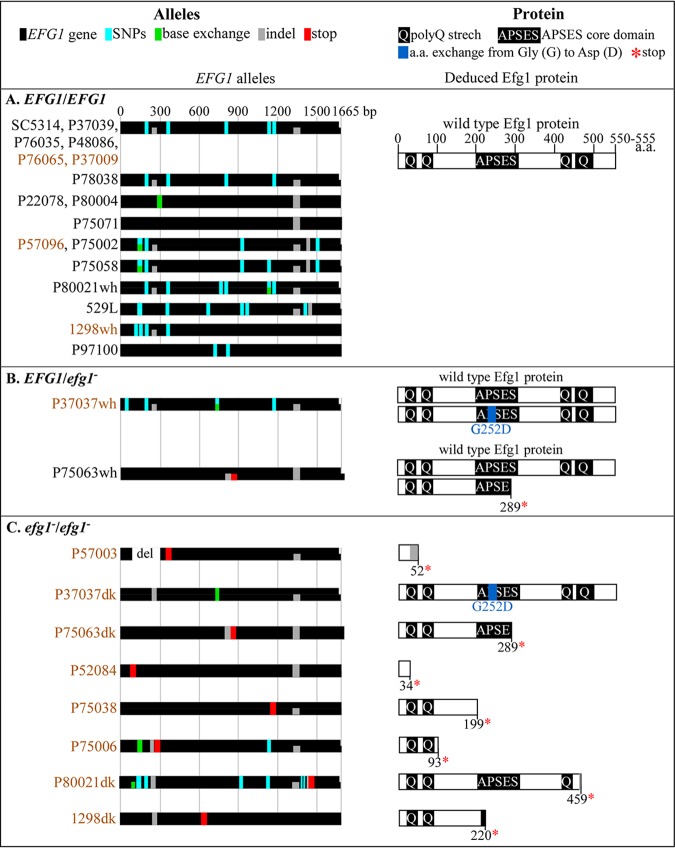
*EFG1* allelic sequences of the 27 **a**/α clinical isolates and the deduced Efg1 proteins. The names of the isolates that did not switch to opaque are black, and those that did switch are brown. (A) Allelic sequences and the deduced proteins of *EFG1/EFG1* isolates. (B) Allelic sequences and the deduced proteins of *EFG1/efg1^−^* isolates. (C) Allelic sequences and deduced proteins of *efg1^−^/efg1^−^* isolates. The keys for color coding and abbreviations are presented at the top of figure. SNPs, single nucleotide polymorphisms; stop, stop codon; base exchange, base exchange resulting in an amino acid (a.a.) exchange compared to the reference sequence (http://www.candidagenome.org/cgi-bin/seqTools); indel, insertion or deletion of base(s). PolyQ stretch, regions rich in glutamine (Q); APSES core domain, DNA binding helix-loop-helix domain which is essential for the function of Efg1; *, protein termination due to stop codon; G252D, amino acid exchange from glycine to aspartic acid.

*EFG1* was sequenced in all 27 clinical isolates. *EFG1* alleles harboring nonsense mutations or a glycine-to-aspartic acid substitution, will be indicated as “*efg1*,” whereas the deletion alleles we generated in strains SC5314 and P37039 will be indicated as “*efg1Δ*.” Of the 27 clinical isolates in the collection, which includes the parent strains SC5314 and P37039, 17 were *EFG1/EFG1*, 2 were *EFG1/efg1*, and 8 were *efg1/efg1* (Table 1). In the key at the top of Fig. 3, the sequenced *EFG1* alleles and the deduced proteins are diagrammed. In the *EFG1/efg1* strain P37037wh, the *efg1* allele harbored a base substitution of aspartic acid for glycine at genomic position 755, resulting in a missense mutation G252D within the APSES core domain (Table 1 and Fig. 3B). In the *EFG1/efg1* strain P35063wh, the *efg1* allele had an insertion of 22 nucleotides at genomic position 849, resulting in a frameshift causing a nonsense mutation at position 289 (Table 1 and Fig. 3B). Seven of the eight *efg1/efg1* strains (P57003, P75063dk, P52084, P75038, P75006, P80021dk, and 1298dk) harbored nonsense mutations caused by point mutations or indel frameshifts, in both *EFG1* alleles (Table 1 and Fig. 3C). A G252D missense mutation in both *EFG1* alleles and partial loss of heterozygosity in strain P37037dk, indicated that it was a derivative of the heterozygous *EFG1/efg1* partner isolate P37037wh (Fig. 3B). The results from sequence analyses supported that conclusion (Fig. 3B and C). Sequence analysis also revealed that P75063dk was derived from P75063wh (Fig. 3B and C).

**TABLE 1:** Origins, clades, and *EFG1* genotypes of the 27 clinical **a**/α isolates and derived deletion mutants analyzed in this study^*a*^

*EFG1* genotype^*b*^	Isolate no.	Strain^*c*^	Body location(s)^*d*^	Country of origin^*e*^	Clade^*f*^ Ca3 (MLST)	*EFG1* alleles^*g*^
*EFG1/EFG1*	1	SC5314	NI	USA	I (1)	*EFG1*/*EFG1*
	2	P37039	HI, Sp	USA	I (1)	*EFG1*/*EFG1*
	3	P76035	BSI	USA	I (1)	*EFG1*/*EFG1*
	4	P78038	BSI	USA	I	*EFG1*/*EFG1*
	5	P48086	BSI	USA	I	*EFG1*/*EFG1*
	6	P76065	BSI	USA	I	*EFG1*/*EFG1*
	7	P22078	BSI	UK	SA	*EFG1*/*EFG1*
	8	P75071	BSI	Italy	SA (4)	*EFG1*/*EFG1*
	9	P80004	BSI	USA	SA (4)	*EFG1*/*EFG1*
	10	P57096	BSI	Brazil	E (11)	*EFG1*/*EFG1*
	11	P75002	BSI	Spain	E	*EFG1*/*EFG1*
	12	P75058	BSI	Switz.	E (11)	*EFG1*/*EFG1*
	13	P80021wh	BSI	Italy	NG (3)	*EFG1*/*EFG1*
	14	529L	OC	UK	NG	*EFG1*/*EFG1*
	15	1298wh	NI	Uganda	NG (18)	*EFG1*/*EFG1*
	16	P37009	HI, Sp	Unknown	NG	*EFG1*/*EFG1*
	17	P97100	BSI	Czech. R	NG (NG)	*EFG1/EFG1*

***EFG1/efg1Δ***		**SC5314**		**USA**	**I (1)**	***EFG1*/*efg1*Δ**
		**P37039**		**USA**	**I (1)**	***EFG1*/*efg1*Δ**

*EFG1/efg1^−^*	18	P37037wh	HI, Sp	USA	I (1)	*EFG1*/*EFG1*(G252D)
	19	P75063wh	BSI	France	SA (4)	*EFG1*/*EFG1*g.849ins22nt (289*)

***efg1Δ/efg1Δ***		**SC5314**		**USA**	**I (1)**	***efg1*Δ/*efg1*Δ**
		**P37039**		**USA**	**I (1)**	***efg1*Δ/*efg1*Δ**

*efg1^−^/efg1^−^*	20	P57003	BSI	USA	I (1)	*EFG1*g.93-298del(52*)/*EFG1*g.93-289del(52*)
	21	P37037dk	HI, Sp	USA	I (1)	*EFG1*(G252D)/*EFG1*(G252D)
	22	P75063dk	BSI	France	SA (4)	*EFG1*g.849ins22nt(289*)/*EFG1*g.849ins22nt(289*)
	23	P52084	BSI	Canada	SA (4)	*EFG1*(Q34*)/*EFG1*(Q34*)
	24	P75038	BSI	Turkey	SA	*EFG1*(Q199*)/*EFG1*(Q199*)
	25	P75006	BSI	Spain	E (11)	*EFG1*(Q93*)/*EFG1*(Q93*)
	26	P80021dk	BSI	Italy	E (11)	*EFG1*g.1361ins1nt(459*)/*EFG1*g.1361ins1nt(459*)
	27	1298dk	NI	Uganda	NG (18)	*EFG1*(Y220*)/*EFG1*(Y220*)

a See Table S1 for the origins of strains noted in references.

b Deletion derivatives generated for strains SC5314 and P37039 (21) are shown in boldface type and were not considered or numbered as part of the basic collection of clinical **a**/α isolates. The wild-type parent strains SC5314 and P37039 were considered members of the collection of the 27 clinical **a**/α isolates. Deletion derivatives are therefore not numbered and are distinguished from natural *EFG1* mutants by a delta symbol.

c Each of four pairs of isolates (P37037wh and P37037dk, P75063wh and P75063dk, P80021wh and P80021dk, and 1298wh and 1298dk) was obtained from one of four individuals.

d NI, not identified: HI, healthy individual; Sp, sputum; BSI, bloodstream; OC, oral cavity.

e USA, United States of America; UK, United Kingdom; Switz., Switzerland; Czech. R, Czech Republic.

f Clades were determined by RFLPs identified by the mid-repeat sequence CA3 (42, 43). See explanation for MLST clades in Table S2 in the supplemental material.

g The numbers after *EFG1* and in parentheses refer to nucleotide positions and amino acid positions, respectively. g, genomic position in *EFG1* gene; del, deletion; ins, insertion; nt, nucleotide; D, aspartic acid; G, glycine; Q, glutamine; *, stop codon; Y, tyrosine; A, adenine; G, guanine; C, cytosine; T, thymine.

*EFG1* polymorphisms of the collection were used to generate a dendrogram of relatedness (see Fig. S1 in the supplemental material), using the neighbor-joining method (26). The *EFG1* sequences of 25 of the 27 isolates separated into three clades, consistent with clades SA (MLST 4), clade E (MLST 11), and I (MLST 1) (Fig. S1). It should be noted that the *efg1/efg1* mutants were distributed among the three major clades (Fig. S1). It should also be noted that the dendrogram generated by the *EFG1* sequences is highly consistent with the groupings of Ca3 fingerprinting and MLST analyses (Fig. S1).

10.1128/mSphere.00795-19.1 FIG S1 A dendrogram of *EFG1* relatedness based on *EFG1* polymorphisms. Clade assignments are presented immediately to the right, based on RFLPs probed with the mid-repeat sequence CA3 and, in parentheses, MLST when available. See Table S2 for explanations about the Ca3 and MLST analysis. The *EFG1* genotypes of the clinical isolates are presented to the far right. The *efg1*^−^ alleles in the isolates are shown in red. Download FIG S1, PDF file, 0.2 MB. Copyright © 2020 Park et al. 2020 Park et al. This content is distributed under the terms of the Creative Commons Attribution 4.0 International license.

### Switching under the eight sets of conditions.

None of the 27 strains in the collection formed either gray or opaque colonies on Gluc-agar (1.25% glucose) under any of the four sets of environmental conditions (25°C and air, 25°C and 5% CO_2_, 37°C and air, and 37°C and 5% CO_2_). However, on GlcNAc-agar, 13 of the 27 strains in the collection (48%) formed opaque colonies under one or more sets of conditions (Fig. 2). Seven of the 13 clinical isolates that switched to opaque formed gray cells, but only at 25°C, as was the case for the *efg1Δ/efg1Δ* derivatives of SC5314 and P37039 (21) (Fig. 1E and 2A). Switching by isolates in each of the three *EFG1* genotypes is dealt with separately in the following sections.

### Switching by *EFG1/EFG1* isolates.

Of the 17 clinical isolates that were *EFG1/EFG1*, four (P76065, P57096, 1298wh, and P37009) underwent switching from white to opaque on GlcNAc-agar under one or more sets of environmental conditions (Fig. 2B and Table S3). P76065 switched to opaque at 25°C in 5% CO_2_ and 37°C in air and 5% CO_2_ (Fig. 2B and Table S3). P57096 switched to opaque in 25°C in air and 5% CO_2_ (Fig. 2B and Table S3). 1298wh and P37009 switched to opaque only in air at 5% CO_2_. All three switching patterns differed from those exhibited by the two *efg1Δ/efg1Δ* derivatives of strains SC5314 and P37039 (Fig. 2A). None of the 17 *EFG1/EFG1* strains, including those that switched to opaque, formed gray cells under any of the four sets of conditions on GlcNAc-agar (Fig. 2B and Table 2). These results suggest that mutations in genes other than *EFG1* resulted in the capacity to switch to opaque and the fact that these mutations did not derepress switching to gray reinforces that suggestion.

**TABLE 2:** Summary of the phenotypic switching characteristics and complementation results for the 27 clinical **a**/α isolates and deletion derivatives of SC5314 and P37039^*a*^

*EFG1* genotype	Isolate no.	Strain	Phenotypic transition^*b*^			Complementation by sc*EFG1*^*c*^		
			Wh to Op	Wh to Gr	Hy	Wh to Op	Wh to Gr	Hy
*EFG1*/*EFG1*	1	SC5314	−	−	+			
	2	P37039	−	−	+			
	3	P76035	−	−	+			
	4	P78038	−	−	−			nt
	5	P48086	−	−	+			
	6	P76065	+	−	+	No		
	7	P22078	−	−	+			
	8	P75071	−	−	+			
	9	P80004	−	−	+			
	10	P57096	+	−	+	No		
	11	P75002	−	−	−			nt
	12	P75058	−	−	−			nt
	13	P80021wh	−	−	+			
	14	529L	−	−	−			nt
	15	1298wh	+	−	+	No		
	16	P37009	+	−	+	No		
	17	P97100	−	−	+			

***EFG1*/*efg1*Δ**		**SC5314**	**−**	**−**	**+**			
		**P37039**	**−**	**−**	**+**			

*EFG1*/*efg1*^−^	18	P37037wh	+	−	+	No		
	19	P75063wh	−	−	+			

***efg1*Δ/*efg1*Δ**		**SC5314**	**+**	**+**	**−**	**Yes**	**Yes**	**Yes**
		**P37039**	**+**	**+**	**−**	**Yes**	**Yes**	**Yes**

*efg1*^−^/*efg1*^−^	20	P57003	+	+	−	Yes	Yes	Yes
	21	P37037dk	+	+	−	Yes	Yes	Yes
	22	P75063dk	+	+	−	Yes	Yes	Yes
	23	P52084	+	+	−	Yes	Yes	Yes
	24	P75038	+	+	−	Yes	Yes	Yes
	25	P75006	+	+	−	Yes	Yes	Yes
	26	P80021dk	+	−	−	No	na	No
	27	1298dk	+	+	−	Yes	Yes	Yes

a Summary of the phenotypic switching characteristics, including gray and hypha formation, and complementation results for the collection of 27 clinical **a**/α isolates and the deletion derivatives of strains SC5314 and P37039. Wh, white; Op, opaque; Gr, gray; Hy, hyphae.

b The white-to-opaque transition (Wh to Op) and the white-to-gray transition (Wh to Gr) was tested on GlcNAc-agar under four sets of environmental conditions (data presented in Fig. 2). The yeast-to-hypha formation transition was tested in suspension and on agar plates, both in the presence of 10% serum.

c Complementation by sc*EFG1* represents reestablishment of repression of the white-to-opaque transition, reestablishment of repression of the white-to-gray transition and reestablishment of hypha induction by serum. nt, not tested; na, not applicable; yes, complemented; no, not complemented.

10.1128/mSphere.00795-19.4 TABLE S3 Switching frequency of the four *EFG1*/*EFG1* clinical isolates that switched to opaque and their *EFG1*/*scEFG1* derivatives generated by site-specific integration at one of the *EFG1* alleles. Switching was assessed on GlcNAc-agar under the four sets of environmental conditions. The switching data represent the means ± standard deviations for three independently performed experiments. Switching includes data for homogenous opaque colonies and opaque sectored colonies in which the sectors contained only opaque cells. These strains did not form gray cells under any of the conditions tested. Download Table S3, DOCX file, 0.02 MB. Copyright © 2020 Park et al. 2020 Park et al. This content is distributed under the terms of the Creative Commons Attribution 4.0 International license.

### Switching by *EFG1/efg1* isolates.

There were only two *EFG1/efg1* strains in the collection, P37037wh and P75063wh. One of these two strains, P37037wh, underwent switching to opaque, but only on GlcNAc-agar at 37°C in 5% CO_2_ (Fig. 2C and Table S4), a response pattern different from those of the 12 other isolates that switched (Fig. 2A, B, and D). This mutant did not form gray cells under any of the four conditions (Table 2). P37037wh harbored one *efg1* allele with an aspartic acid substitution for glycine (Table 1 and Fig. 3). These results suggest that just as in the case of the *EFG1/EFG1* strains that switched to opaque, repression was due to a mutation in one or more genes other than *EFG1*. This conclusion was supported by the fact that the two generated *EFG1/efg1Δ* derivatives of SC5314 and P37039 did not switch (Fig. 2A and Table S4).

10.1128/mSphere.00795-19.5 TABLE S4 Switching frequency of white to opaque for the *efg1*Δ/*efg1*Δ mutants, *efg1*^−^*/efg1*^−^ natural strains, and their wild type and *efg1*^−^/sc*EFG1* complemented derivatives generated by site-specific integration of one of the *EFG1* alleles. The switching was assessed on GlcNAc-agar under the four sets of environmental conditions. The switching data represent the means ± standard deviations for three independently performed experiments. These data include only switching from white to opaque (homogenous opaque colonies and colonies with homogenous opaque sectors); they do not include switching from white to gray. Download Table S4, DOCX file, 0.02 MB. Copyright © 2020 Park et al. 2020 Park et al. This content is distributed under the terms of the Creative Commons Attribution 4.0 International license.

### Switching by *efg1*/*efg1* isolates.

All eight of the clinical isolates that were *efg1*/*efg1* underwent switching from white to opaque on GlcNAc-agar under more than one of the four sets of conditions on GlcNAc-agar (Fig. 2D and Table S4). Seven of the eight underwent switching to gray and opaque in response to the four sets of environmental conditions (Fig. 2D and Table S4) in a fashion highly similar to that of the *efg1*Δ/*efg1*Δ deletion derivatives of SC5314 and P37039 (Fig. 2A and Table S4). Only one of the eight clinical *efg1*/*efg1* strains, P80021dk, that did not switch to gray (Fig. 2D and Table 2) exhibited a switching pattern under the four sets of conditions (Fig. 2D and Table S4) that differed from that of the two *efg1*Δ/*efg1*Δ derivatives (Fig. 2A), but it was highly similar to that of the *EFG1/EFG1* strain P57096 (Fig. 2B), suggesting that the mutation was in the same gene in the two clinical isolates, even though the former was *EFG1/EFG1* and the latter was *efg1*/*efg1*. Another explanation is that the deduced domains remaining in the truncated P80021dk Efg1 protein resulted in partial function.

### Complementation of *EFG1* mutants.

To test whether the repression of switching by **a**/α clinical isolates could be reinstated (i.e., complemented) by reintroducing a functional copy of *EFG1*, we performed site-specific integration at the native *EFG1* locus of one copy of *EFG1* from strain SC5314 (“sc*EFG1*”), under the control of the native promoter (21). As we previously demonstrated (21), reintroducing a copy of sc*EFG1* into the SC5314 *efg1*Δ/*efg1*Δ and P37039 *efg1*Δ/*efg1*Δ derivatives, under the control of the wild-type *EFG1* promoter, reinstated switching repression. Site-specific integration of one copy of sc*EFG1* in the four *EFG1/EFG1* strains that switched to opaque, generating strains P76065 *EFG1*/sc*EFG1*, P57096 *EFG1*/sc*EFG1*, 1298wh *EFG1*/sc*EFG1*, and P37009 *EFG1*/sc*EFG1* (Table S1) did not reinstate repression of switching (i.e., did not result in complementation) in any of the four strains (Tables 2, 3, and S3). These results support the conclusion that derepression of switching in these four clinical *EFG1/EFG1* isolates is due to mutations in one or more repressor genes other than *EFG1*. We next tested whether integration of sc*EFG1* complemented the one clinical *EFG1/efg1* mutant, P37037wh, which switched to opaque. It did not (Tables 2, 3, and S4), again indicating that a mutation in a gene other than *EFG1* derepressed switching to opaque. Finally, we tested whether the addition of one copy of sc*EFG1* complemented the seven clinical *efg1*/*efg1* strains that switched to gray and opaque and the one *efg1*/*efg1* strain (P80021dk) that switched to opaque but not gray. The addition of sc*EFG1* reestablished the repression of switching (i.e., complementation) in the seven strains that switched to opaque and gray (Fig. 2D and Tables 2 and 3). However, the site-specific integration of sc*EFG1* into P37037dk, generating P37037dk *efg1*/sc*EFG1*, did not repress switching to opaque and repressed switching to gray (Tables 2, 3, and S4), as was the case for P37037wh (Fig. 2 and Table S4). Site-specific integration of sc*EFG1* in P80021dk did not restore repression of switching, indicating a mutation in a repressor gene other than *EFG1*. These results support the conclusion that seven of the eight *efg1*/*efg1* strains, which represent 30% of the entire collection of clinical isolates, switched to gray and opaque due to *efg1* mutations and that only one *efg1*/*efg1* strain carried a second mutation that derepressed switching to opaque.

**TABLE 3:** Results of complementation experiments^*a*^

Category	Strain	Switching^*b*^			
		25°C		37°C	
		Air	5% CO_2_	Air	5% CO_2_
Controls	SC5314 *efg1*Δ/*efg1*Δ	(+)	(++++)	(++++)	++++
	SC5314 *efg1*Δ/sc*EFG1*	−	−	−	−
					
	P37039 *efg1*Δ/*efg1*Δ	−	(++++)	(++++)	++++
	P37039 *efg1*Δ/sc*EFG1*	−	−	−	−

*EFG1/EFG1*	P76065 *EFG1/EFG1*	−	++++	++	++++
	P76065 *EFG1/*sc*EFG1*	−	++++	++	+++
					
	P57096 *EFG1/EFG1*	+++	++++	−	−
	P57096 *EFG1/*sc*EFG1*	++++	++++	−	−
					
	1298wh *EFG1/EFG1*	−	++++	−	−
	1298wh *EFG1/*sc*EFG1*	−	++++	−	−
					
	P37009 *EFG1/EFG1*	−	++	−	−
	P37009 *EFG1/* sc*EFG1*	−	++	−	−

*EFG1/efg1*^−^	P37037wh *EFG1/efg1*^−^	−	−	−	+++
	P37037wh *EFG1/*sc*EFG1*	−	−	−	++++

*efg1*^−^/*efg1*^−^	P57003 *efg1*^−^/*efg1*^−^	−	(++++)	(++++)	++++
	P57003 *efg1*^−^/sc*EFG1*	−	−	−	−
					
	P37037dk *efg1*^−^/*efg1*^−^	(+)	(++++)	(++++)	++++
	P37037dk *efg1*^−^/sc*EFG1*	−	−	−	++++
					
	P75063dk *efg1*^−^/*efg1*^−^	−	(++++)	(++++)	++++
	P75063dk *efg1*^−^/sc*EFG1*	−	−	−	−
					
	P52084 *efg1*^−^/*efg1*^−^	−	(++++)	(++++)	++++
	P52084 *efg1*^−^/sc*EFG1*	−	−	−	−
					
	P75038 *efg1*^−^/*efg1*^−^	−	(++++)	(++++)	++++
	P75038 *efg1*^−^/sc*EFG1*	−	−	−	−
					
	P75006 *efg1*^−^/*efg1*^−^	−	(++++)	(++++)	++++
	P75006 *efg1*^−^/sc*EFG1*	−	−	−	−
					
	P80021dk *efg1*^−^/*efg1*^−^	++++	++++	−	−
	*P80021dk efg1^−^/scEFG1*	++++	++++	−	−
					
	1298dk *efg1*^−^/*efg1*^−^	(++)	(++++)	(++++)	++++
	1298dk *efg1*^−^/sc*EFG1*	−	−	−	−

a Results of complementation experiments, in which one copy of SC5314 *EFG1* (sc*EFG1*) was inserted into the native locus at one allele, under the control of native promoter, by site-specific integration in strains that switched to opaque. See Tables S3 and S4 for switching frequencies. Switching analyses were performed on GlcNAc-agar.

b ++++, 80 to 100% opaque colonies; +++, 21 to 80% opaque colonies; ++, 11 to 20% opaque colonies; −, no opaque or mixed colonies. Parentheses indicates colonies with mixed cellular phenotypes. At 25°C in air or 5% CO_2_, the mixtures include a majority of opaque cells and a minority of white and gray cells; at 37°C in air, the mixtures include a majority of opaque cells and a minority of white cells (no gray cells). (++++), 80 to 100%; (++), 11 to 20%; (+), 1 to 10%. Each assessment represents the results of pooled data for three independent experiments. The total number of colonies ranged between 425 and 2,059.

### Hypha formation.

**FIG 4:**
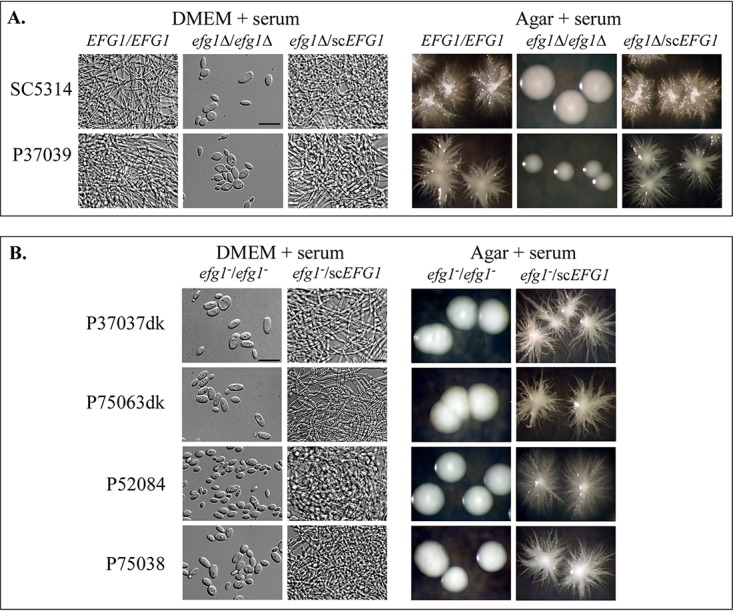
Induction of hypha formation in clinical **a**/α strains and their complemented derivatives. Hypha induction was tested in suspension cultures in DMEM medium plus 10% serum (DMEM + serum), and on nonnutrient agar containing 10% serum (Agar + serum) at 37°C in 5% CO_2_. Complementation was tested by the addition of a single copy of sc*EFG1* through site-specific integration. (A) Cellular phenotypes and colony phenotypes under hypha-inducing conditions for SC5314 and P37039 *EFG1/EFG1*, their *efg1*Δ/*efg1*Δ derivatives, and the complemented *efg1*Δ/sc*EFG1* derivatives. (B) Examples of the phenotypes of *efg1*/*efg1* strains and the complemented *efg1^−^/*sc*EFG1* derivatives. Bars, 10 μm.

*EFG1* not only plays a negative regulatory role in white-opaque switching (10, 27), but it also plays a positive regulatory role in hypha formation (28, 29). Serum induced hypha formation in the *EFG1/EFG1* control strains SC5314 and P37039, but not in the deletion derivatives SC5314 *efg1*Δ/*efg1*Δ and P37039 *efg1*Δ/*efg1*Δ (Fig. 4A and Table 2). Site-specific integration of a copy of sc*EFG1*, generating strains SC5314 *efg1Δ*/sc*EFG1* and P37039 *efg1Δ*/sc*EFG1*, reestablished hypha induction (i.e., caused complementation) (Fig. 4A and Table 2). Serum induced hypha formation in 13 of the 17 *EFG1/EFG1* isolates (Table 2). Most notably, serum induced hypha formation in all four of the *EFG1/EFG1* strains that underwent white-opaque switching (Table 2), supporting the conclusion that mutations in genes other than *EFG1* were responsible for the derepression of switching in those strains. The four *EFG1/EFG1* strains that did not form hyphae also did not undergo the white-to-opaque transition (Table 2), suggesting that a mutation in a gene other than *EFG1* was responsible for the lack of hypha induction. Both *EFG1*/*efg1* clinical isolates formed hyphae, as was the case for the *EFG1/efg1Δ* derivatives of strains SC5314 and P37039 (Table 2). Seven of the eight *efg1^−^*/*efg1* clinical isolates, which switched to opaque and gray, did not form hypha, which was the case for the *efg1Δ/efg1Δ* derivatives of SC5314 and P37039 (Table 2). Representative micrographs of four of these isolates (P37037dk, P75063dk, P52084, and P75038) are presented in Fig. 4B. In seven of the eight *efg1*/*efg1* isolates, for which site-specific integration of sc*EFG1* reestablished the repression of switching, the addition of sc*EFG1* also reestablished serum-induced hypha formation (Table 2 and Fig. 4B). The one *efg1*/*efg1* isolate, P80021dk, which switched to opaque but not gray, and for which the addition of sc*EFG1* does not complement switching, was also not complemented for hypha formation by the addition of sc*EFG1* (Table 2). These results support the conclusion that, in the case of seven of the eight *efg1*/*efg1* isolates, the mutation in *EFG1* was solely responsible for the derepression of switching and the repression of hypha formation. In the case of one *efg1*/*efg1* isolate, P80021dk, a mutation in a gene in addition to the mutation in *EFG1* repressed switching and the induction of hypha formation.

### Intestinal colonization.

**FIG 5:**
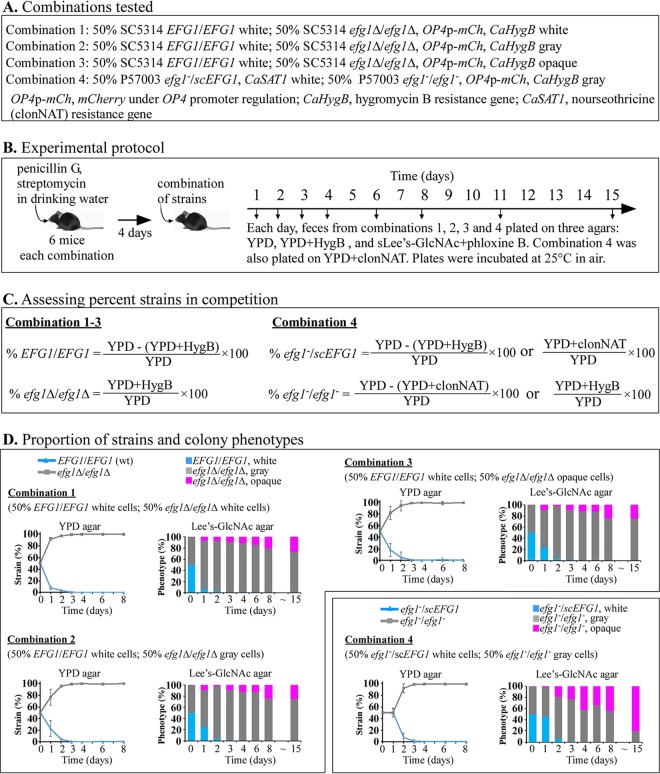
Competition experiments between SC5314 *EFG1/EFG1* strain and *efg1Δ/efg1Δ* derivatives and between the complemented P57003 *efg1/scEFG1 efg1/efg1* strain in the mouse model of gastrointestinal colonization. (A) Cell phenotype and genotypes of the pairs of strains in each of the four tested combinations. (B) Experimental protocol used to analyze colonizing populations. (C) Calculations for assessing the proportions of the two strains in each combination. (D) Proportion of each of the two strains and the proportion of the colony phenotypes in the feces over a 15-day period following ingestion.

Previous studies have demonstrated that *efg1/efg1* strains outcompete *EFG1/EFG1* strains in mouse gastrointestinal (GI) colonization models (23, 24, 30, 31). Liang et al. (23) further demonstrated that in competition experiments in which fecal samples were plated on CHROMagar over time and incubated in air at 22°C for 5 days, the colonizing *efg1Δ*/*efg1Δ* or *efg1/efg1* cells formed colonies containing almost exclusively gray cells after 2 days postingestion. However, in an *in vitro* study, we found that cells of *efg1Δ*/*efg1Δ* derivatives of strains SC5314 and P37039 that were plated on GlcNAc-agar and incubated at 25°C formed gray cells, but not opaque cells, and at 37°C, they formed opaque cells, but not gray cells (21). Hence, ingested cells in the GI tract may not be capable of expressing the gray phenotype at the site of colonization because of the physiological temperature (37°C). Hence, plating assays performed at 25°C or 22°C may induce opaque cells to switch back to gray. We therefore considered the possibility that the proportion of colony phenotypes assessed after 5 days on CHROMagar Candida, a glucose-based agar containing a “chromogenis mix” (32), in air at 22°C (23), may not have accurately reflected the actual phenotypes of progenitor cells at the site of colonization. We performed similar competition experiments between SC5314 *EFG1/EFG1* versus SC5314 *efg1*Δ/*efg1*Δ cells (50:50) and between P57003 *efg1*/sc*EFG1* versus P57003 *efg1/efg1* cells (50:50) in which we plated fecal samples over time on GlcNAc-agar and incubated the plates for 5 days at 25°C in air, conditions we previously found stabilized both the opaque and gray phenotype but did not induce the opaque phenotype (21). The genotypes and cell phenotypes of the initial strains in the four competition experiments are presented in Fig. 5A, the experimental protocol is shown in Fig. 5B, the methods for computing the proportions of initial strains over time are shown in Fig. 5C, and the results are shown in Fig. 5D. The *efg1Δ*/*efg1Δ* strain outcompeted the *EFG1/EFG1* strain in combinations 1, 2, and 3, and the *efg1*/*efg1* strain outcompeted the *efg1*/sc*EFG1* strain in combination 4. For all four combinations, the homozygous mutant strains represented more than 90% of the colonizing populations after 2 days, and close to 100% after 3 days postingestion (Fig. 5D), results similar to those of Liang et al. (23). For all four combinations, the gray phenotype dominated after 2 days postingestion and remained dominant through 8 days postingestion, again consistent with the results of Liang et al. (23). However, the proportion of opaque cells differed between our study and that of Liang et al. (23) as time progressed. Whereas Liang et al. (23) found that in competition experiments between **a**/α *EFG1/EFG1* and *efg1*/*efg1* cells, the colonizing populations did not form **a**/α opaque colonies 10 or more days postingestion, we found here a significant proportion of opaque colonies, approximately 25% of the colonies from feces in combinations 1, 2, and 3 and 50% in combination 4 after 8 days (Fig. 5E). After 15 days, the proportions increased to 30, 30, 30, and 80% (Fig. 5E).

**FIG 6:**
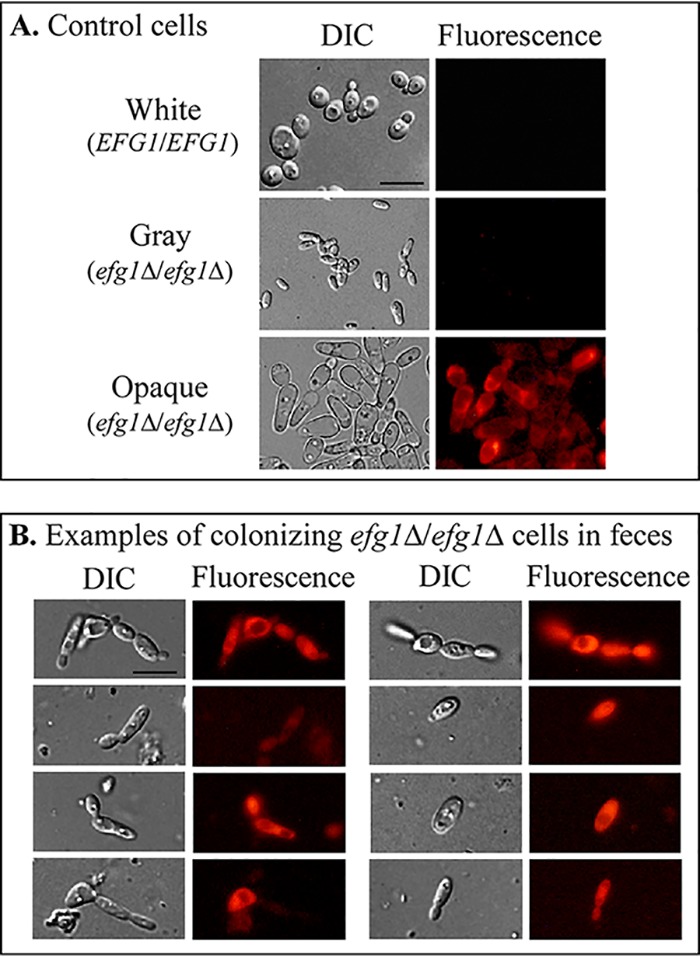
The majority of live cells in 8 day fecal samples of mice that ingested a combination of 50% SC5314 *EFG1/EFG1* white cells and 50% SC5314 *efg1Δ/efg1Δ*, *OP4*p-mCh white cells exhibited almost exclusively opaque cell morphologies and expressed *mCherry*, which was under the regulation of the native opaque-specific *OP4* promoter, indicating that opaque was the dominating phenotype in the colonizing cell population. Cell morphology was assessed by DIC microscopy and expression of *mCherry* by fluorescence microscopy. (A) Cell morphologies and *mCherry* expression of *EFG1/EFG1* white cells, *efg1Δ*/*efg1Δ* gray cells, and *efg1Δ*/*efg1Δ* opaque cells generated *in vitro*. (B) Cell morphologies and *mCherry* expression by cells colonizing the feces 8 days after ingestion of the 50:50 mixture of SC5314 *EFG1/EFG1* white cells and *efg1*Δ/*efg1*Δ *OP4*p-mCh white cells (combination 1 in Fig. 5A). Of 100 cells analyzed, more than 90% exhibited the distinct opaque phase phenotype, and more than 70% expressed *mCherry* throughout their cytoplasm. Bars, 10 μm.

We considered the possibility that the differences in the proportions of opaque cells observed in our study versus those of Liang et al. (23) may be due to differences in the agar media. Agar based on supplemented Lee’s medium (25) and containing GlcNAc was employed in our plating study, whereas glucose-based CHROMagar Candida medium (32) was used in the study by Liang et al. (23). Our major concern was that the incubation conditions for assessing phenotype in both of the studies may have in fact influenced phenotype, and therefore, may not have accurately produced results that reflected the actual proportions of cellular phenotypes at the site of colonization. We therefore examined the phenotypes of live cells directly in feces in combination 1 (Fig. 5A) after 8 days postingestion. Feces were simply diluted, and the live C. albicans cells were viewed microscopically. Since the colonizing SC5314 *efg1Δ/efg1Δ* cells harbored the gene for *mCherry* under the control of the promoter of the opaque-specific gene *OP4* (12, 33) (Table S1), the live cells were examined by phase-contrast microscopy for morphology and by fluorescence microscopy for opaque-specific *mCherry* expression. White and gray cells of SC5314 *efg1Δ/efg1Δ* do not express *OP4* and therefore do not fluoresce, whereas opaque cells express *OP4* and therefore fluoresce throughout their cytoplasm (Fig. 6A). Of 100 cells examined directly in fecal samples, more than 90% exhibited the elongate opaque, not gray, cell morphology, and more than 70% exhibited mCherry fluorescence (Fig. 6B). These results indicate that the majority of *efg1Δ/efg1Δ* cells colonizing the feces after 8 days postingestion express the opaque, not gray, phenotype, and therefore, that the results of the plating experiments performed here (Fig. 5E) and previously (23) may not accurately reflect the actual cellular phenotype of *efg1*/*efg1* cells at the site of GI colonization.

## DISCUSSION

Xie et al. (20) first reported that approximately one third of clinical isolates, which are predominately **a**/α (18, 19), can be induced to switch from white to opaque by plating white cells on GlcNAc-agar and incubating the plates at 25°C in 5% CO_2_. We have found here, using a broader screen that included eight combinations of environmental conditions, that close to half of a collection of 27 clinical **a**/α isolates could be induced to switch. Xie et al. (20) further demonstrated that deletion of any one of three transcription factor genes, *RFG1*, *BRG1*, or *EFG1*, derepressed white-to-opaque switching in an **a**/α strain that did not switch. Park et al. (21) subsequently reported the same for deletion of *SFL2*. Liang et al. (23) further reported that 6 of 31 clinical isolates (19%) harbored hemizygous or homozygous *EFG1* mutations and that homozygous *EFG1* mutants switched to the gray phenotype, which was previously shown to exhibit unique characteristics and to be part of a triphasic switching system (22). We report here that 8 of the 27 clinical **a**/α isolates (30%) were *efg1*/*efg1* deletion mutants and that 7 of the 8 could switch to gray and all 8 could switch to opaque (21). However, we also found that four *EFG1/EFG1* isolates and one *EFG1/efg1* isolate (19%) could switch, but as a result of a mutation in a gene other than *EFG1*.

### *EFG1* and white-to-opaque switching.

It has been almost 20 years since Sonneborn et al. (10) first demonstrated that Efg1 suppresses switching in *MTL* homozygous strains of C. albicans. Subsequent studies then revealed that *EFG1* null mutants outcompeted *EFG1/EFG1* cells in mouse models of gastrointestinal (GI) colonization (24, 34). The results of Tao et al. (22) and Liang et al. (23), and the results presented here, further support these previous studies that suggest that *EFG1* plays a role in GI colonization. The results reported here further demonstrate that 30% of a collection of 27 clinical **a**/α isolates were *efg1*/*efg1*. All eight *efg1*/*efg1* strains in our study underwent white-to-opaque switching, and seven of the eight were complemented for white-opaque switching by reintroducing a functional copy of *EFG1*. Surprisingly, we also found that 4 of the 17 *EFG1/EFG1* **a**/α strains and 1 of the 2 *EFG1*/*efg1* **a**/α strains in the tested collection underwent white-to-opaque switching and that switching in none of these strains was complemented by the addition of a functional copy of *EFG1*. Together, these results suggest that while half of the **a**/α strains that switch do so as a result of mutations in both *EFG1* alleles, the other half that switch do so due to mutations in genes other than *EFG1*.

### *EFG1* and white-to-gray switching.

Tao et al. (22) and Liang et al. (23) have provided strong evidence that the gray cell phenotype is distinct from the white and opaque cell phenotypes, in morphology, gene expression, and virulence. However, we demonstrated (21) that switching to gray cells could be induced *in vitro* at 25°C, but not at 37°C, and at the single cell level, that individual tiny elongate, gray cells could be induced to morph phenotypically to the larger opaque, pimpled cell morphology prior to cell division. In doing so, they pass smoothly through a morphology that is intermediate between the tiny elongate morphology and the final opaque morphology (21). In support of the contention that gray does represent a unique phenotype (22, 23), we found that the four *EFG1/EFG1* clinical **a**/α isolates, the one *EFG1*/*efg1* clinical **a**/α isolate, and the one *efg1*/*efg1* clinical **a**/α isolate that switched to opaque but could not be complemented for white-opaque switching by site-specific integration of sc*EFG1* could also not be induced to form gray cells, suggesting that there are genes other than *EFG1* that, when mutated, derepress opaque cell formation, but not gray cell formation.

### *EFG1*, switching, and hypha formation.

Opaque cells share several characteristics with hyphae, most notably an elongate shape, an enlarged vacuole, surface antigens (4, 6), a number of regulatory genes (10, 35, 36), and downregulation of the white-specific gene (*WH11*) (37). There are, however, a number of differences, including budding patterns (4), wall ultrastructure, most notably the formation of opaque cell wall pimples (7), the release of extracellular membrane-bound vesicles (7), and the expression of opaque-specific genes, like *OP4* (12, 13, 38). Here, we have presented evidence that a majority of **a**/α *EFG1*/*EFG1* clinical isolates that can be induced to form hyphae cannot be induced to switch, and a majority of the *efg1*/*efg1* clinical isolates that cannot be induced to form hyphae, can be induced to switch. However, there are exceptions to this yin-yang pattern, indicating the existence of additional repressor genes, other than *EFG1*, specific for switching and additional activator genes, other than *EFG1*, specific for hypha formation.

### Gastrointestinal colonization.

Earlier studies demonstrated that *EFG1* deletion mutants outcompete wild-type *EFG1/EFG1* cells in competition experiments in a mouse GI model for colonization (24, 30, 31). Liang et al. (23) recently reported that in such competition experiments, the colonizing *efg1*/*efg1* cells express almost exclusively the gray phenotype. Liang et al. (23) employed a plating assay on CHROMagar, which contains glucose as the sugar source and a proprietary chromogenic mix (32). We performed similar competition experiments, also using a plating assay to assess the phenotype of colonizing cells, employing GlcNAc rather than glucose as the sugar source. In both studies, the temperature (22°C and 25°C, respectively) was far below the physiological temperature. We previously demonstrated (21) that this agar at 25°C in air stabilized the opaque phenotype of **a**/α cells induced *in vitro*. Here, we found that regardless of the initial phenotype of the *efg1Δ/efg1Δ* cells (white, gray, or opaque) in combination with *EFG1/EFG1* white cells, the *efg1Δ/efg1Δ* cells rapidly dominated colonization in the mouse GI colonization model. Also, in the case of the combination of *efg1*/sc*EFG1* white cells and *efg1*/*efg1* gray cells, the *efg1*/*efg1* cells dominated. These results were consistent with those of Liang et al. (23). However, Liang et al. (23) found almost exclusively gray colony formation by the colonizing cells, whereas we found that by 15 days postingestion, a third of the colonizing cells were opaque in combinations 1, 2, and 3, and more than three quarters were opaque in combination 4. This difference between the two studies, combined with our earlier observation (21) that the gray phenotype is not expressed at physiological temperature *in vitro* (21), led us to question the accuracy of both the plating procedure of Liang et al. (23) and that of our study in assessing the phenotypes of the cells in feces. We considered the possibility that both assay procedures, which include 5 days of incubation on an agar substrate in air at temperatures well below the physiological temperature may influence phenotype. We therefore directly examined microscopically the phenotype(s) of live cells in fresh feces. We found that more than 90% of the cells in feces exhibited the opaque cell morphology and more than 70% expressed *mCherry* under the regulation of an opaque-specific promoter. Our results suggest, therefore, that plating experiments may not accurately reflect the phenotype of cells colonizing the GI tract, and that opaque, not gray, may be the dominant phenotype at the site of colonization. The results of Pande et al. (31) are also pertinent to our finding. They found that overexpressing *WOR1* (*WOR1*^OE^) in **a**/α cells causes a competitive advantage over wild-type **a**/α cells in the mouse GI colonization model and that the colonizing *WOR1*^OE^ cells formed opaque-like cells without wall pimples that were unstable on glucose-based agar at 25°C (31). These results support the general conclusion that **a**/α strains that can switch, regardless of the switching repressor or a switching activator gene that is mutated, outcompete cells that cannot switch in the mouse GI colonization model, and add weight to the conclusion that an opaque or opaque-like phenotype is expressed at the site of GI colonization.

### Final comments.

Our results indicate that, as previously reported, a third or more of clinical isolates, which are mainly **a**/α, can undergo the white-to-opaque transition. Half of these strains switch due to mutations in the repressor *EFG1*, while half switch due to mutations in other repressor genes. Since the *efg1*/*efg1* **a**/α strains outcompete wild-type *EFG1* strains in the mouse GI model, they appear to have an advantage in GI colonization, and this advantage appears to be accompanied by expression of the opaque phenotype. On the other hand, the *efg1*/*efg1* strains may lose invasiveness since they do not appear to form hyphae. If true, one might consider the possibility that opaque *efg1*/*efg1* cells may serve to colonize the GI tract as a commensal but not function as an opportunistic pathogen. However, seven of the eight *efg1*/*efg1* isolates that can switch from white to opaque and that cannot be induced to form hyphae were isolated from the bloodstream, suggesting invasiveness and opportunism. This apparent contradiction warrants further investigation.

## MATERIALS AND METHODS

### Strains and media.

The C. albicans strains used in this study are described in Table 1 and Table S1 in the supplemental material. The strains were maintained at room temperature on agar containing YPD medium (1% yeast extract, 2% peptone, 2% glucose). Escherichia coli strain XL1-Blue (Agilent Technologies, TX, USA), used to generate and maintain plasmids, was grown in LB medium (1% tryptone, 0.5% yeast extract, 1% NaCl) plus 100 μg/ml ampicillin.

### Plasmids and construction of C. albicans strains.

The plasmid pEFG1C (21), which contains a wild-type copy of *EFG1* obtained from strain SC5314 (sc*EFG1*), was used to test for complementation by site-specific integration at the *EFG1* locus. A pmCherry-HygB plasmid (21) was employed to create strains harboring the *mCherry* gene under the regulation of the opaque-specific *OP4* promoter. For selection of cells transformed with the gene insertion cassettes from the plasmids above, YPD agar containing 200 μg/ml of nourseothricin or 1 mg/ml of hygromycin B was used, depending on the selection marker. The C. albicans *SAT1* (*CaSAT1*) gene was used to confer resistance to nourseothricin, and the *CaHygB* gene was used to confer resistance to hygromycin B. Integration into proper loci was verified by PCR. The genotypes of the constructed strains are provided in Table S1.

### Sequence analysis.

To sequence *EFG1* in the 27 clinical isolates, we prepared genomic DNA from strains grown in 5 ml of a YPD suspension culture as described previously (39). Genomic DNA was used as a template for PCR amplification with the primer pairs EFG1-5´F/-3´R (Table S5). Phusion high-fidelity DNA polymerase (New England Biolabs) was employed to amplify the genes by PCR. The amplified DNA fragments of *EFG1* were sequenced by Sanger sequencing with the primers EFG1-seq1 through EFG1-seq5 (Table S5).

10.1128/mSphere.00795-19.6 TABLE S5 Primers used in this study. Download Table S5, DOCX file, 0.02 MB. Copyright © 2020 Park et al. 2020 Park et al. This content is distributed under the terms of the Creative Commons Attribution 4.0 International license.

### Dendrogram for *EFG1* relatedness.

Polymorphisms along the *EFG1* sequences were translated into allelic frequencies, with 1.0 for the presence of a homozygous polymorphism, 0.5 for a heterozygous polymorphism, and 0.0 for the absence of a polymorphism. Allelic frequencies were used to generate Nei’s pairwise genetic distances (40) with the GENDIST program of the PHYLIP package, version 3.695 (http://evolution.genetics.washington.edu/phylip.html). The unrooted dendrogram (see Fig. S1 in the supplemental material) was generated by the neighbor-joining method (26) implemented in the NEIGHBOR program of the PHYLIP package.

### Switching assay.

Switching was tested on agar containing 1.25% glucose (Gluc-agar) or 2.00% GlcNAc (GlcNAc-agar), with the agar containing supplemented Lee’s medium (sLee’s) (25, 41) and 5 μg/liter of phloxine B, which stains opaque colonies or opaque sectors pink to red (6). Eight sets of environmental conditions were tested, which included all combinatorial permutations of sugar source (1.25% glucose versus 2% GlcNAc), temperature (25°C versus 37°C), and atmosphere (air [0.04% CO_2_] versus 5% CO_2_). Cells from YPD suspension cultures were grown overnight (∼20 h) at 25°C and then plated on Gluc-agar or GlcNAc-agar plates. Colony phenotypes were analyzed after 5 days of incubation. Homogeneous opaque colonies containing exclusively opaque cells and white colonies with opaque sectors with the latter containing exclusively opaque cells were counted as opaque. Representative colonies were analyzed for cellular phenotypes as described in a previous study (21). In all assays, more than 10 colonies were assessed microscopically for white, opaque, and gray cells. Switching experiments were repeated at least three times, and in some cases, the data were pooled. A total of at least 500 colonies were analyzed for each strain under each condition.

### Hypha formation assays.

Hypha formation was assessed in two ways, in suspension cultures containing Dulbecco’s modified Eagle’s medium (DMEM) supplemented with 10% fetal calf serum (FCS) in 24-well plates and incubated at 37°C in 5% CO_2_ for 24 h or by plating on nonnutrient agar containing 10% serum and incubating at 37°C in 5% CO_2_ for 3 days.

### *MTL* genotypes of a/α opaque cells.

Cells from at least six individual opaque colonies of each strain of the clinical **a**/α collection were genotyped for the *MTL* configuration by PCR with the two primer pairs, *MTL***a**1F/R and *MTL*α2F/R (Table S5).

### Immunolocalization of the opaque-specific pimple marker.

Rabbit-derived polyclonal antipimple antiserum against an opaque pimple antigen (7) was used to visualize the formation of opaque-specific pimples by immunocytochemistry. Opaque cells were heat killed in a 65°C water bath for 1 h, pelleted, and resuspended in phosphate-buffered solution (PBS) supplemented with 10% normal goat serum to block nonspecific binding. A 1:50 dilution of rabbit serum was preabsorbed five times with heat-killed homozygous *MTL* white cells to remove antibodies to surface antigens common to white and opaque cells (7). After staining with the primary antiserum, cells were washed with PBS and treated with Alexa Fluor 488-tagged goat anti-rabbit secondary antibody (Jackson ImmunoResearch, West Grove, PA). Fluorescent images were captured using a Leica TCS SP8 confocal microscope, and all images were similarly processed with Image J software.

### Imaging colonies and cells.

Colonies grown on agar plates were imaged through a stereo microscope equipped with a Nikon E990 digital camera. Differential interference contrast (DIC) and fluorescence microscopic images of cells expressing *mCherry* were obtained with a Canon Rebel T3i digital camera attached to a Nikon-PE2000 inverted microscope through a 60× plan water immersion objective. To image cells directly in fecal samples, fecal pellets were mixed in distilled H_2_O at a dilution of 1:10. Cells were imaged by phase-contrast and fluorescence microscopy.

### Mouse gastrointestinal tract colonization.

All procedures complied with regulatory guidelines defined by the Iowa University IACUC committee. C57BL/6J (Jackson Laboratory) female mice 6 to 7 weeks old were treated with 1 mg/ml of penicillin G and 2 mg/ml of streptomycin in their drinking water for 4 days. Mice were orally inoculated with 0.2 ml (3 × 10^7^ cells) of a cell combination (Fig. 5A) in PBS. Six mice were tested for each cell mixture. To assess the levels of colonization, competition, and phenotype switching, fresh fecal samples were collected postingestion at time intervals. Each fecal sample was homogenized in 0.2 ml of water, and dilutions of the fecal samples were plated on YPD agar plates, YPD+nourseothricin agar (YPD+clonNAT), YPD+hygromycin B agar plates (YPD+HygB), and GlcNAc-agar plates (Fig. 5B). All agar media contained 50 μg/ml of chloramphenicol. Cultures were incubated at 25°C in air for 3 to 6 days, and colony phenotypes were assessed (Fig. 5B). GlcNAc-agar plates were used to determine phenotypic switching to opaque, since this agar medium maintains the cellular phenotype of *efg1Δ/efg1Δ* and *efg1*/*efg1* mutants and does not induce switching to opaque or gray when incubation is performed at 25°C in air (21). The data from six mice were pooled for each combination of cells at each time point.
