# Supplementary material for: EFG1 Mutations, Phenotypic Switching, and Colonization by Clinical a/α Strains of Candida albicans
Source: mSphere. 2020 Feb 5;5(1):e00795-19. doi: 10.1128/mSphere.00795-19 (PMC7002308; doi:10.1128/mSphere.00795-19)
Supplement: TABLE S1 [file mSphere.00795-19-st001.docx]

| Strain | Parental strain | Genotype | Reference |
| --- | --- | --- | --- |
| SC5314 | - | See Table 1 | (1) |
| P37039 | - | See Table 1 | (2) |
| SC5314 *efg1*Δ/*efg1*Δ | SC5314 | *efg1*::*FRT*/*efg1*::*FRT* | (3) |
| P37039 *efg1*Δ/*efg1*Δ | P37039 | *efg1*::*FRT*/*efg1*::*FRT* | (3) |
| SC5314 *efg1*Δ/*efg1*Δ-mChH | SC5314 *efg1*Δ/*efg1*Δ | *efg1*::*FRT*/*efg1*::*FRT*, *OP4*/*op4*::*OP4p*-*mCherry*-*CaHygB* | (3) |
| P37039 *efg1*Δ/*efg1*Δ-mChH | P37039 *efg1*Δ/*efg1*Δ | *efg1*::*FRT*/*efg1*::*FRT*, *OP4*/*op4*::*OP4p*-*mCherry*-*CaHygB* | (3) |
| P76035 | - | See Table 1 | (4) |
| P78038 | - | See Table 1 | (4) |
| P48086 | - | See Table 1 | (4) |
| P57003 | - | See Table 1 | (4) |
| P37037wh | - | See Table 1 | (2) |
| P37037dk | - | See Table 1 | (2) |
| P76065 | - | See Table 1 | (4) |
| P22078 | - | See Table 1 | (4) |
| P75071 | - | See Table 1 | (4) |
| P75063wh | - | See Table 1 | (4) |
| P75063dk | - | See Table 1 | (4) |
| P52084 | - | See Table 1 | (4) |
| P75038 | - | See Table 1 | (5) |
| P80004 | - | See Table 1 | (4) |
| P57096 | - | See Table 1 | (4) |
| P75002 | - | See Table 1 | (4) |
| P75058 | - | See Table 1 | (4) |
| P75006 | - | See Table 1 | (4) |
| P80021wh | - | See Table 1 | (4) |
| P80021dk | - | See Table 1 | (4) |
| 529L | - | See Table 1 | (6) |
| 1298wh | - | See Table 1 | This study |
| 1298dk | - | See Table 1 | This study |
| P37009 | - | See Table 1 | This study |
| P97100 | - | See Table 1 | (5) |
| SC5314  *efg1*Δ/sc*EFG1* | SC5314 *efg1*Δ/*efg1*Δ | *efg1*::*FRT*/*efg1*::*scEFG1*-*CaSAT1* | This study |
| P37039  *efg1*Δ/sc*EFG1* | P37039 *efg1*Δ/*efg1*Δ | *efg1*::*FRT*/*efg1*::*scEFG1*-*CaSAT1* | This study |
| P57003  *efg1*^−^/sc*EFG1* | P57003 | P57003*EFG1*/P57003*EFG1*::*scEFG1*-*CaSAT1* | This study |
| P37037dk  *efg1*^−^/sc*EFG1* | P37037dk | P37037dk*EFG1*(755G>A)/P37037dk*EFG1*(755G>A)::*scEFG1*-*CaSAT1* | This study |
| P37037wh  *EFG1*/sc*EFG1* | P37037wh | P37037wh/P37037dk*EFG1*(755G>A)::*scEFG1*-*CaSAT1* | This study |
| P75063dk  *efg1*^−^/sc*EFG1* | P75063dk | P75063dk*EFG1*/P75063dk*EFG1*::*scEFG1*-*CaSAT1* | This study |
| P75038  *efg1*^−^/sc*EFG1* | P75038 | P75038*EFG1*/P75038*EFG1*::*scEFG1*-*CaSAT1* | This study |
| P52084  *efg1*^−^/sc*EFG1* | P52084 | P52084*EFG1*/P52084*EFG1*::*scEFG1*-*CaSAT1* | This study |
| P75006  *efg1*^−^/sc*EFG1* | P75006 | P75006*EFG1*/P75006*EFG1*::*scEFG1*-*CaSAT1* | This study |
| P80021dk  *efg1*^−^/sc*EFG1* | P80021dk | P80021dk*EFG1*/P80021dk*EFG1*::*scEFG1*-*CaSAT1* | This study |
| 1298dk  *efg1*^−^/sc*EFG1* | 1298dk | 1298dk*EFG1*/1298dk*EFG1*::*scEFG1*-*CaSAT1* | This study |
| P76065  *EFG1*/sc*EFG1* | P76065 | P76065*EFG1*/P76065*EFG1*::*scEFG1*-*CaSAT1* | This study |
| P57096  *EFG1*/sc*EFG1* | P57096 | P57096*EFG1*/P57096*EFG1*::*scEFG1*-*CaSAT1* | This study |
| 1298wh  *EFG1*/sc*EFG1* | 1298wh | 1298wh*EFG1*/1298wh*EFG1*::*scEFG1*-*CaSAT1* | This study |
| P37009  *EFG1*/sc*EFG1* | P37009 | P37009*EFG1*/P37009*EFG1*::*scEFG1*-*CaSAT1* | This study |
| P57003-mChH | P57003 | *OP4*/*op4*::*OP4p*-*mCherry*-*CaHygB* | This study |
| P37037dk-mChH | P37037dk | *OP4*/*op4*::*OP4p*-*mCherry*-*CaHygB* | This study |
| P75063dk-mChH | P75063dk | *OP4*/*op4*::*OP4p*-*mCherry*-*CaHygB* | This study |
| P52084-mChH | P52084 | *OP4*/*op4*::*OP4p*-*mCherry*-*CaHygB* | This study |
| P75038-mChH | P75038 | *OP4*/*op4*::*OP4p*-*mCherry*-*CaHygB* | This study |
| P75006-mChH | P75006 | *OP4*/*op4*::*OP4p*-*mCherry*-*CaHygB* | This study |
| P80021dk-mChH | P80021dk | *OP4*/*op4*::*OP4p*-*mCherry*-*CaHygB* | This study |
| 1298dk-mChH | 1298dk | *OP4*/*op4*::*OP4p*-*mCherry*-*CaHygB* | This study |
|  |  |  |  |

sc*EFG1*, a copy of *EFG1* gene from SC5314 strain; G>A, nucleotide exchange from guanine to adenine.

**REFERENCES**

1. Gillum, A.M., Tsay, E.Y., and Kirsch, D.R. (1984). Isolation of the Candida albicans gene for orotidine-5'-phosphate decarboxylase by complementation of S. cerevisiae ura3 and E. coli pyrF mutations. Molecular & general genetics : MGG *198*, 179-182.
2. Pujol, C., Messer, S.A., Pfaller, M., and Soll, D.R. (2003). Drug resistance is not directly affected by mating type locus zygosity in Candida albicans. Antimicrob Agents Chemother *47*, 1207-1212.
3. Park, Y.N., Conway, K., Conway, T.P., Daniels, K.J., and Soll, D.R. (2019). Roles of the Transcription Factors Sfl2 and Efg1 in White-Opaque Switching in a/alpha Strains of Candida albicans. mSphere *4*.
4. Pujol, C., Pfaller, M., and Soll, D.R. (2002). Ca3 fingerprinting of Candida albicans bloodstream isolates from the United States, Canada, South America, and Europe reveals a European clade. J Clin Microbiol *40*, 2729-2740.
5. Pfaller, M.A., Diekema, D.J., Rinaldi, M.G., Barnes, R., Hu, B., Veselov, A.V., Tiraboschi, N., Nagy, E., and Gibbs, D.L. (2005). Results from the ARTEMIS DISK Global Antifungal Surveillance Study: a 6.5-year analysis of susceptibilities of Candida and other yeast species to fluconazole and voriconazole by standardized disk diffusion testing. J Clin Microbiol *43*, 5848-5859.
6. Rahman, D., Mistry, M., Thavaraj, S., Challacombe, S.J., and Naglik, J.R. (2007). Murine model of concurrent oral and vaginal Candida albicans colonization to study epithelial host-pathogen interactions. Microbes Infect *9*, 615-622.
