# Supplementary material for: EFG1 Mutations, Phenotypic Switching, and Colonization by Clinical a/α Strains of Candida albicans
Source: mSphere. 2020 Feb 5;5(1):e00795-19. doi: 10.1128/mSphere.00795-19 (PMC7002308; doi:10.1128/mSphere.00795-19)
Supplement: TABLE S2 [file mSphere.00795-19-st002.docx]

|  | Ca3 clade | MLST clade | Consensus |
| --- | --- | --- | --- |
| P75038 | SA | (NA) | clade SA_(MLST 4)_ |
| P22078 | SA | (NA) | clade SA_(MLST 4)_ |
| P80004 | SA | 4 | clade SA_(MLST 4)_ |
| P75071 | SA | 4 | clade SA_(MLST 4)_ |
| P75063wh | SA | 4 | clade SA_(MLST 4)_ |
| P75063dk | SA | 4 | clade SA_(MLST 4)_ |
| P97100 | SA | (NA) | clade SA_(MLST 4)_ |
| P52084 | SA | 4 | clade SA_(MLST 4)_ |
|  |  |  |  |
| P80021wh | (NA) | 3 | clade III_(MLST 3)_ |
|  |  |  |  |
| 1298dk | (NA) | 18 | NG_(MLST 18)_ |
| 1298wh | (NA) | 18 | NG_(MLST 18)_ |
|  |  |  |  |
| P75058 | E | 11 | clade E_(MLST 11)_ |
| P57096 | E | 11 | clade E_(MLST 11)_ |
| P75002 | E | (NA) | clade E_(MLST 11)_ |
| P80021dk | E | 11 | clade E_(MLST 11)_ |
| P75006 | E | 11 | clade E_(MLST 11)_ |
|  |  |  |  |
| P78038 | I | (NA) | clade I_(MLST 1)_ |
| P37037wh | I | 1 | clade I_(MLST 1)_ |
| P37037dk | I | 1 | clade I_(MLST 1)_ |
| P57003 | I | 1 | clade I_(MLST 1)_ |
| P76035 | I | 1 | clade I_(MLST 1)_ |
| P76065 | I | (NA) | clade I_(MLST 1)_ |
| P37039 | I | 1 | clade I_(MLST 1)_ |
| P48086 | I | (NA) | clade I_(MLST 1)_ |
| P37009 | I | (NA) | clade I_(MLST 1)_ |
| SC5314 | I | 1 | clade I_(MLST 1)_ |
|  |  |  |  |
| 529L | (NA) | Not 1,2,3,4,8,9,11 or 18 | NG_(MLST NG)_ |
|  |  |  |  |

(NA). isolate not analyzed with this method

NG. Isolate not clustering with any of the major clades
