# Supplementary figures and images for: EFG1 Mutations, Phenotypic Switching, and Colonization by Clinical a/α Strains of Candida albicans
Source: mSphere. 2020 Feb 5;5(1):e00795-19. doi: 10.1128/mSphere.00795-19 (PMC7002308; doi:10.1128/mSphere.00795-19)

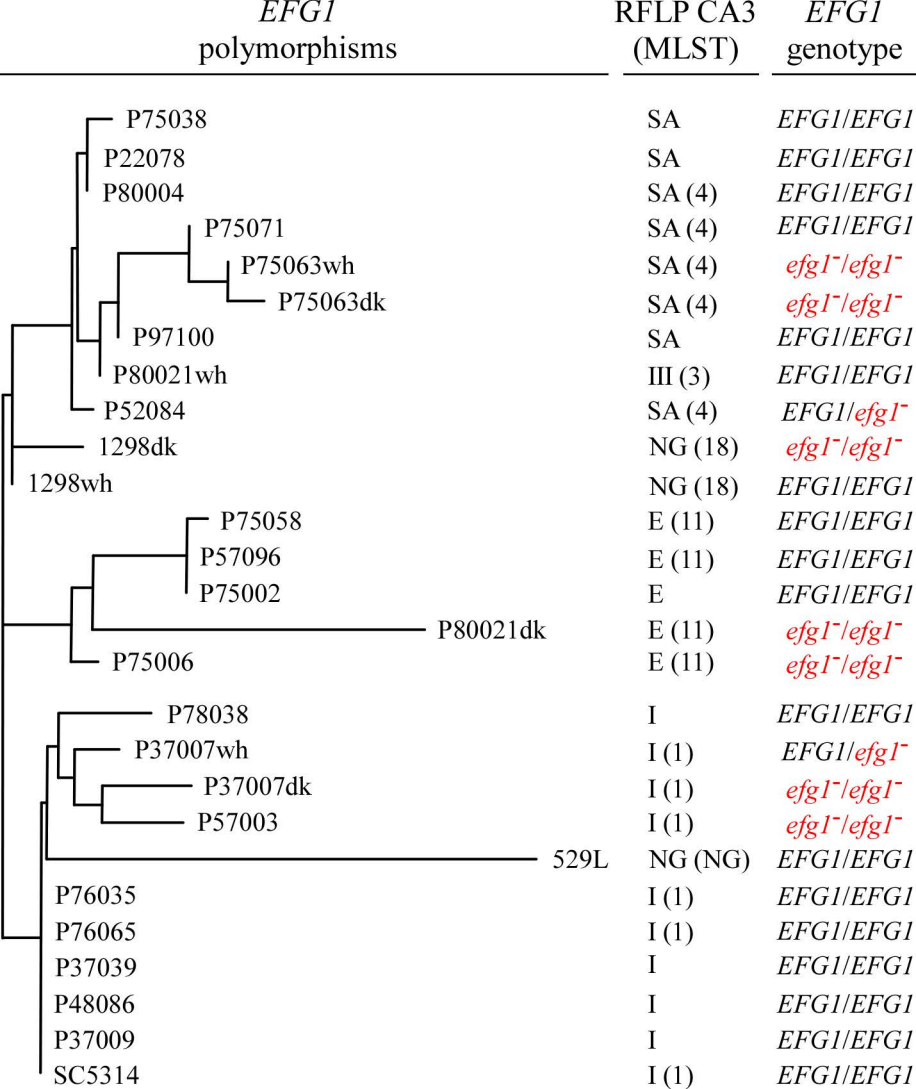

Supplement: FIG S1 [file mSphere.00795-19-sf001.pdf]
