# Supplementary material for: EFG1 Mutations, Phenotypic Switching, and Colonization by Clinical a/α Strains of Candida albicans
Source: mSphere. 2020 Feb 5;5(1):e00795-19. doi: 10.1128/mSphere.00795-19 (PMC7002308; doi:10.1128/mSphere.00795-19)
Supplement: TABLE S3 [file mSphere.00795-19-st003.docx]

|  | 25°C, air | | 25°C, 5% CO_2_ | | 37°C, air | | 37°C, 5% CO_2_ | |
| --- | --- | --- | --- | --- | --- | --- | --- | --- |
|  | Total col. no. | Switching frequency (%) | Total col. no. | Switching frequency (%) | Total col. no. | Switching frequency (%) | Total col. no. | Switching frequency (%) |
|  |  |  |  |  |  |  |  |  |
| P75065 *EFG1/EFG1* | 599 | 0.8 ± 0.7 | 898 | 98.0 ± 3.5 | 712 | 15.3 ± 18.2 | 482 | 88.5 ± 9.5 |
| P75065 *EFG1/*sc*EFG1* | 909 | 0.2 ± 0.4 | 812 | 99.9 ± 0.2 | 718 | 11.3 ± 2.0 | 654 | 77.8 ± 6.2 |
|  |  |  |  |  |  |  |  |  |
| P57096 *EFG1/EFG1* | 1092 | 73.9 ± 9.7 | 1135 | 100 | 975 | < 0.10 | 933 | 0.1 ± 0.1 |
| P57096 *EFG1/*sc*EFG1* | 988 | 60.2 ± 4.1 | 838 | 100 | 796 | < 0.13 | 711 | < 0.14 |
|  |  |  |  |  |  |  |  |  |
| 1298wh *EFG1/EFG1* | 2252 | < 0.04 | 2198 | 99.9 ± 0.3 | 2367 | < 0.04 | 2002 | < 0.05 |
| 1298wh *EFG1/*sc*EFG1* | 1783 | < 0.06 | 1748 | 98.4 ± 2.1 | 1746 | < 0.06 | 1734 | < 0.06 |
|  |  |  |  |  |  |  |  |  |
| P37009 *EFG1/EFG1* | 1059 | < 0.09 | 1039 | 15.4 ± 3.5 | 1122 | < 0.09 | 1023 | < 0.10 |
| P37009 *EFG1/*sc*EFG1* | 978 | < 0.10 | 1002 | 14.1 ± 4.1 | 997 | < 0.10 | 968 | < 0.10 |
|  |  |  |  |  |  |  |  |  |

Total col. no., total colony number.
