# Supplementary material for: EFG1 Mutations, Phenotypic Switching, and Colonization by Clinical a/α Strains of Candida albicans
Source: mSphere. 2020 Feb 5;5(1):e00795-19. doi: 10.1128/mSphere.00795-19 (PMC7002308; doi:10.1128/mSphere.00795-19)
Supplement: TABLE S4 [file mSphere.00795-19-st004.docx]

|  | 25°C, air | | 25°C, 5% CO_2_ | | 37°C, air | | 37°C, 5% CO_2_ | |
| --- | --- | --- | --- | --- | --- | --- | --- | --- |
| Strains | Total col. no. | Switching frequency (%)^a^ | Total col. no. | Switching frequency (%)^b^ | Total col. no. | Switching frequency (%)^c^ | Total col. no. | Switching frequency (%) |
|  |  |  |  |  |  |  |  |  |
| SC5314 *EFG1/EFG1* | 1328 | < 0.08 | 1179 | < 0.08 | 1212 | < 0.08 | 1028 | < 0.10 |
| SC5314 *EFG1*/*efg1*Δ | 911 | < 0.11 | 866 | < 0.12 | 988 | < 0.10 | 1088 | < 0.09 |
| SC5314 *efg1*Δ/*efg1*Δ | 1523 | 4.6 ± 5.3 | 1661 | 2.5 ± 3.1 | 1647 | 100 | 1545 | 100 |
| SC5314 *efg1*Δ/sc*EFG1* | 1140 | < 0.09 | 1097 | < 0.09 | 1055 | < 0.09 | 1107 | < 0.09 |
|  |  |  |  |  |  |  |  |  |
| P37039 *EFG1/EFG1* | 1259 | < 0.08 | 1420 | < 0.07 | 1306 | < 0.08 | 1293 | < 0.8 |
| P37039 *EFG1*/*efg1*Δ | 984 | < 0.10 | 941 | < 0.11 | 966 | < 0.10 | 950 | < 0.11 |
| P37039 *efg1*Δ/*efg1*Δ | 1095 | < 0.09 | 993 | 1.0 ± 1.0 | 1031 | 0.2 ± 0.3 | 1116 | 95.8 ± 7.2 |
| P37039 *efg1*Δ/sc*EFG1* | 1173 | < 0.09 | 1029 | < 0.10 | 992 | < 0.10 | 1047 | < 0.10 |
|  |  |  |  |  |  |  |  |  |
| P57003 *efg1^−^/efg1^−^* | 1223 | 6.9 ± 6.6 | 1273 | 100 | 1372 | 1.0 ± 0.2 | 1062 | 100 |
| P57003 *efg1^−^*/sc*EFG1* | 1843 | < 0.07 | 1847 | < 0.07 | 1717 | < 0.09 | 1326 | < 0.08 |
|  |  |  |  |  |  |  |  |  |
| P37037wh *EFG1*/*efg1^−^* | 2059 | < 0.05 | 1965 | 0.8 ± 1.3 | 1963 | < 0.05 | 1879 | 85.5 ± 12.2 |
| P37037wh *EFG1*/*scEFG1* | 1096 | < 0.09 | 978 | < 0.10 | 898 | < 0.11 | 1033 | 62.3 ± 4.2 |
| P37037dk *efg1^−^/efg1^−^* | 499 | < 0.2 | 490 | 100 | 425 | 100 | 502 | 100 |
| P37037dk *efg1^−^*/sc*EFG1* | 1365 | < 0.05 | 1394 | < 0.05 | 1151 | < 0.05 | 1332 | 80.4 ± 6.0 |
|  |  |  |  |  |  |  |  |  |
| P75063wh *EFG1*/*efg1^−^* | 942 | < 0.11 | 1370 | < 0.07 | 1251 | < 0.08 | 1189 | < 0.08 |
| P75063dk *efg1^−^/efg1^−^* | 504 | < 0.2 | 582 | 100 | 470 | 100 | 577 | 100 |
| P75063dk *efg1^−^*/sc*EFG1* | 1096 | < 0.09 | 978 | < 0.10 | 898 | < 0.11 | 1033 | < 0.10 |
|  |  |  |  |  |  |  |  |  |
| P52084 *efg1^−^/efg1^−^* | 945 | 0.4 ± 0.3 | 943 | 100 | 832 | 100 | 912 | 100 |
| P52084 *efg1^−^*/sc*EFG1* | 1045 | < 0.10 | 993 | < 0.10 | 1027 | < 0.10 | 788 | < 0.13 |
|  |  |  |  |  |  |  |  |  |
| P75038 *efg1^−^/efg1^−^* | 1388 | < 0.07 | 1540 | 0.4 ± 0.6 | 1340 | 0.1 ± 0.2 | 1426 | 100 |
| P75038 *efg1^−^*/sc*EFG1* | 665 | < 0.15 | 670 | < 0.15 | 613 | < 0.16 | 543 | < 0.18 |
|  |  |  |  |  |  |  |  |  |
| P75006 *efg1^−^/efg1^−^* | 824 | < 0.07 | 943 | 0.1 ± 0.2 | 934 | 100 | 877 | 99.9 ± 0.2 |
| P75006 *efg1^−^*/sc*EFG1* | 641 | < 0.16 | 668 | < 0.15 | 701 | < 0.14 | 577 | < 0.17 |
|  |  |  |  |  |  |  |  |  |
| P80021dk *efg1^−^/efg1^−^* | 631 | 99.5 ± 0.5 | 605 | 100 | 602 | < 0.17 | 598 | < 0.17 |
| P80021dk *efg1^−^*/sc*EFG1* | 649 | 99.9 ± 0.2 | 547 | 98.5 ± 1.0 | 506 | < 0.2 | 646 | < 0.15 |
|  |  |  |  |  |  |  |  |  |
| 1298dk *efg1^−^/efg1^−^* | 1529 | 22 ± 18.1 | 1593 | 0.3 ± 0.5 | 1515 | 100 | 1483 | 100 |
| 1298dk *efg1^−^*/sc*EFG1* | 1783 | < 0.06 | 1755 | < 0.06 | 1852 | < 0.05 | 1587 | < 0.06 |
|  |  |  |  |  |  |  |  |  |

^a^: Non-opaque colonies of *efg1*Δ/*efg1*Δ and *efg1^−^/efg1^−^* strains contained a mixture of white cells and a majority of gray cells.

^b^: Non-opaque colonies of *efg1*Δ/*efg1*Δ and *efg1^−^/efg1^−^* strains contained a mixture of a minority of white and gray cells, and a majority of opaque (≥ 50%) cells.

^c^: Non-opaque colonies of *efg1*Δ/*efg1*Δ and *efg1^−^/efg1^−^* strains contained a mixture of white cells and a majority of opaque cells.

Percent switching refers to the proportion of colonies fully opaque or with opaque sectors. Total col. no., total colony number; sc*EFG1*, an *EFG1* copy obtained from SC5314 strain and integrated into genomic locus of *EFG1* in the strains.
