# Supplementary material for: EFG1 Mutations, Phenotypic Switching, and Colonization by Clinical a/α Strains of Candida albicans
Source: mSphere. 2020 Feb 5;5(1):e00795-19. doi: 10.1128/mSphere.00795-19 (PMC7002308; doi:10.1128/mSphere.00795-19)
Supplement: TABLE S5 [file mSphere.00795-19-st005.docx]

| **Primer** | **Sequence (5´ to 3´)** |
| --- | --- |
| EFG1-5´F | GAGAACAAAAGAAGGGCCCATTATTCATTGCAC |
| EFG1-3´R | TTGTACCTTCCGCGGTAGACGCTTACTGCTTGC |
| EFG1-seq1 | AACAACCAACCAACCCTTAACCC |
| EFG1-seq2 | CCACATGGTAGTTGTTACTCGTGG |
| EFG1-seq3 | CTACCAGGTCAACAAGCAGTACC |
| EFG1-seq4 | TTGAATGAAGGAAACTTTCCAATC |
| EFG1-seq5 | CACTACTGCTGGTACCCCTCAAGG |
| MTLa1F | TTGAAGCGTGAGAGGCAGGAG |
| MTLa1R | GATTAGGCTGTTTGTTCTTCTCG |
| MTLα2F | CATGAATTCACGTCTGGAGGCAC |
| MTLα2R | AAGCAGCCAACTCAGGTCAC |
